# Atypical presentations of CNS tuberculosis: A case series from South India in a high-burden, resource-limited setting

**DOI:** 10.1016/j.idcr.2025.e02454

**Published:** 2025-12-08

**Authors:** Parthiban Palaniappan, Sivakumar K

**Affiliations:** a Department of General Medicine, Coimbatore Medical College Hospital, Coimbatore, Tamil Nadu, India; b Department of General Medicine, Coimbatore Medical College Hospital, Coimbatore, Tamil Nadu, India

**Keywords:** CNS tuberculosis, Tuberculoma, Low-resource settings, Atypical presentation, Neurocysticercosis

## Abstract

**Background:**

Central nervous system (CNS) tuberculosis is one of the most severe extrapulmonary manifestations of TB, associated with high morbidity and mortality if not diagnosed and treated early. In India, which bears the highest global burden of TB, the challenge is compounded by resource limitations. Even in apex government medical colleges serving millions, advanced imaging such as Magnetic Resonance Imaging (MRI) may take several days, and molecular or histopathological confirmation is often delayed. In such settings, clinicians must rely on high clinical suspicion and pragmatic use of available resources.

**Case presentations:**

We describe six patients with atypical manifestations of CNS tuberculosis encountered at a government medical college hospital in South India. Presentations included parenchymal tuberculomas, extraparenchymal lesions, and radiological mimics of neoplasm and demyelinating disease. Diagnostic challenges were heightened by limited access to advanced imaging and delays in confirmatory testing. Management strategies were tailored to available resources, combining antitubercular therapy with steroids and supportive interventions. Clinical outcomes varied, with some patients demonstrating marked improvement, while others had residual neurological deficits.

**Conclusion:**

This case series underscores the protean nature of CNS tuberculosis and the diagnostic dilemmas it creates in resource-constrained settings. Awareness of atypical presentations and timely initiation of empirical therapy, even in the absence of definitive imaging or laboratory confirmation, can be lifesaving. Our experience highlights the importance of clinical acumen and decision-making in high-burden, low-resource environments where delays in investigation are common.

## Highlights

• Early empirical antitubercular therapy can be lifesaving in CNS tuberculosis.
• Corticosteroids, osmotic therapy, and seizure control improve outcomes.
• CT, fundus examination, and CSF analysis are vital in resource-limited settings.
• Atypical presentations mimic stroke, epilepsy, or tumors, risking misdiagnosis.
• Vigilance and pragmatic strategies reduce morbidity in high-burden regions.

## Introduction

Tuberculosis (TB) remains a major global health problem, with India contributing nearly one-fourth of all cases worldwide [1]. Central nervous system (CNS) TB, though less frequent than pulmonary disease, carries disproportionately high morbidity and mortality when diagnosis and treatment are delayed [2]. Government medical colleges in India serve as apex referral centres for millions, but even here diagnostic capacity is overstretched. A contrast-enhanced MRI may be delayed for days. Molecular assays such as GeneXpert are available but have low sensitivity in CNS specimens — around 46 % for tuberculous meningitis (TBM) — and cannot be relied on as standalone tests [3]. Thus, clinicians must rely on high suspicion, basic investigations, and clinical judgment. The clinical spectrum of CNS TB is broad. While classical meningitis is well described, atypical forms such as parenchymal tuberculomas, abscesses, and spinal disease often mimic neoplasms, demyelinating disorders, or other infections [4], [5]. Such mimicking presentations create diagnostic dilemmas, particularly in low- and middle-income countries (LMICs). Case reports and series remain critical for sharing practical insights from real-world resource-limited contexts. Here, we present six patients with atypical CNS TB from a South Indian medical college hospital, highlighting diverse presentations, resource-related challenges, and lessons for early recognition and management.

## Case description

### Case 1 – The ring that lied

A 13-year-old girl, the daughter of a migrant textile worker from Bihar, presented to our hospital in Tamil Nadu with a four-month history of fever and weight loss, followed by one month of headache and diplopia due to left lateral rectus palsy. An MRI performed in Bihar suggested neurocysticercosis (NCC), and she was referred for further evaluation [6], [7].

On admission, her presentation appeared atypical for NCC. Fundus examination revealed bilateral choroidal tubercles, strongly suggestive of tuberculosis. A lumbar puncture was performed, and cerebrospinal fluid (CSF) was sent for cytology, biochemistry, ADA, Gram stain, AFB smear, culture, CBNAAT, and Taenia solium IgG. She was empirically started on steroids and mannitol for raised intracranial pressure. High-Resolution CT (HRCT) chest revealed a left apical cavitary lesion with adjacent tree-in-bud nodules consistent with active pulmonary tuberculosis, without radiological evidence of miliary nodules, indicating that her disseminated CNS involvement occurred despite the absence of miliary lung disease. A leg radiograph showed no calcified parasitic cysts.

**Fig. 1:**
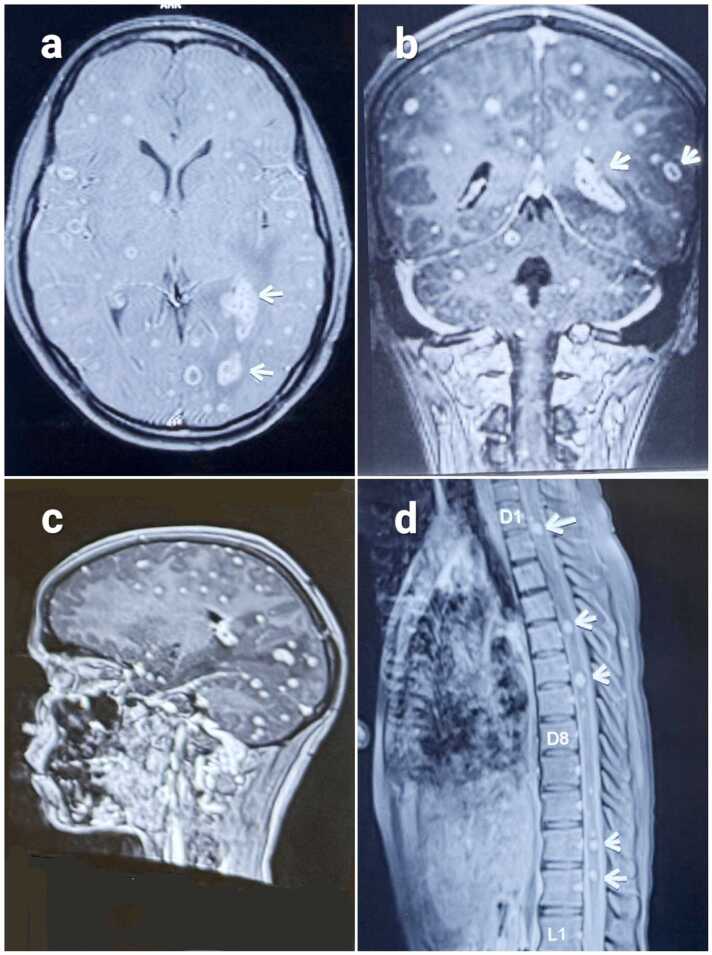
Contrast-enhanced MRI brain and spine showing multiple ring-enhancing lesions. (a) Axial post-contrast T1-weighted image demonstrates multiple ring-enhancing lesions in the bilateral cerebral hemispheres and arrow shows Left Temporal Conglomerate lesion. (b) Coronal post-contrast T1-weighted image shows enhancing lesions involving the cerebral parenchyma, arrow shows Conglomerate lesion. (c) Sagittal post-contrast T1-weighted image reveals numerous enhancing lesions scattered throughout the supratentorial and infratentorial compartments. (d) Sagittal post-contrast T1-weighted spine image depicts multiple enhancing nodular lesions along the dorsal spinal cord (arrows).

CSF analysis on Day 2 showed lymphocytic pleocytosis, glucose 37 mg/dL, elevated ADA (19.2 U/L) and a cobweb coagulum on prolonged standing (Fig. 4a). CBNAAT was negative on Day 3, but antitubercular therapy (ATT) was initiated empirically. On Day 4, CSF IgG for *Taenia solium* was negative. MRI brain taken on Day 5, demonstrated multiple ring-enhancing lesions in brain and spinal cord (Fig. 1d), including a left temporal conglomerate with surrounding edema and diffusion (Fig. 1a-c). MR spectroscopy showed elevated choline and lipid peaks, lipid indicating caseation and choline reflecting inflammatory membrane turnover, supporting a tuberculous etiology. CSF culture remained negative. HIV testing was negative.

Her clinical course was initially favourable: by Day 6, headache improved, and by Day 10 her lateral rectus palsy had resolved. However, on Day 23 she developed intractable seizures and decerebrate posturing. An emergency CT revealed coning due to acute herniation, likely secondary to rupture of a tubercle with worsening edema. Despite ICU care, she died on Day 27. The late deterioration was likely due to rupture of a miliary tubercle with acute edema and herniation, a known complication in disseminated CNS TB despite initial radiological and clinical improvement.

**Final diagnosis:** Miliary tuberculosis with CNS tuberculomas, initially misdiagnosed as neurocysticercosis.

**Clinical pearl:** In endemic regions, not all ring-enhancing lesions represent neurocysticercosis (NCC). Fundus examination and early CSF analysis may provide decisive clues, and vigilant monitoring is essential even when initial improvement is seen.

### Case 2 – Paradoxical CNS TB on ATT

A 22-year-old woman with childhood-onset epilepsy since age 12, with no identifiable cause and maintained on sodium valproate with occasional breakthrough seizures every 6–12 months, had been diagnosed with pulmonary tuberculosis (PTB) three months earlier following symptoms of evening fever and weight loss. At that time, she had no neurological features, and CNS involvement was not suspected. Having just completed the intensive phase of first-line ATT a few days prior, she now presented with new generalised tonic–clonic seizures.

On admission, possibilities considered were breakthrough seizures due to epilepsy versus paradoxical CNS tuberculosis [8], [9].

CT brain on Day 1 showed a mild hypodensity in the right parietal region. She was started on mannitol and optimized on antiepileptics. On Day 2, CSF revealed lymphocytic pleocytosis, low glucose (46 mg/dL), and elevated ADA (16 U/L); CBNAAT was negative. HIV testing was negative. Chest imaging showed no evidence of pulmonary TB.

Corticosteroids were added in view of probable TB meningitis. By Day 6, MRI revealed multiple ring-enhancing tuberculomas, predominantly in the right parietal lobe. ATT was continued with regimen modified for CNS TB.

She improved steadily; seizures were well controlled by Day 10, and she was discharged on WHO recommended CNS tuberculosis regimen ATT (2HRZE followed by 10HR) [10], tapering steroids, and regular antiepileptics. At 1-month follow-up, she remained seizure-free with stable neurological status.

**Final diagnosis:** Paradoxical CNS tuberculomas in a patient with PTB on ATT and pre-existing epilepsy.

**Clinical pearl:** New-onset seizures in a patient already on ATT should prompt consideration of paradoxical CNS tuberculosis, even in the absence of HIV or immunosuppression.

### Case 3 – The tumor mimicker

A 61-year-old man, a chronic smoker and alcoholic, presented with 10 days of headache, vomiting, and altered sensorium. There was no history of fever, seizures, or weight loss.

CT brain showed bilateral multiple hypodense white matter lesions, raising suspicion of metastatic disease from an unknown primary [11], [12]. He was empirically started on mannitol and dexamethasone. However, fundus examination revealed bilateral choroidal tubercles, raising suspicion of tuberculosis.

**Fig. 2:**
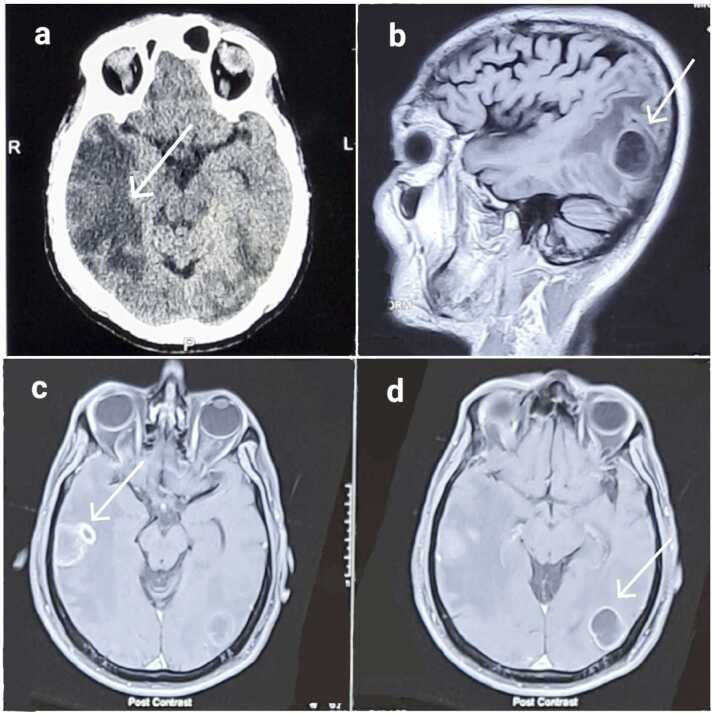
Neuroimaging of tuberculomas of Case 3 (a) Axial non-contrast CT brain showing a hypodense lesion in the right parietal region (arrow) and in left parieto-occipital region. (b) Sagittal T1-weighted MRI (non-contrast) demonstrating a right parietal lesion suggestive of tuberculoma (arrow). (c) Axial post-contrast T1-weighted MRI showing ring-enhancing right parietal tuberculoma with surrounding edema (arrow). (d) Axial post-contrast T1-weighted MRI showing ring-enhancing left parietal tuberculoma (arrow).

CSF analysis on Day 3 revealed lymphocytic pleocytosis, low glucose (42 mg/dL), elevated ADA (18 U/L), and negative CBNAAT. MRI on Day 6 demonstrated multiple ring-enhancing lesions with surrounding edema, consistent with tuberculomas (Fig. 2a-d). Given the ring-enhancing morphology and surrounding edema, a tuberculous abscess was considered in the differential, although overall features favored tuberculoma. HIV testing was negative. Chest imaging showed no evidence of pulmonary TB. Systemic evaluation revealed no evidence of malignancy, and culture remained negative.

With ATT and corticosteroids, his neurological status improved; by Day 10 he was discharged clinically stable with no residual impairment of consciousness. At 2-month follow-up, he remained clinically stable with no recurrent symptoms.

**Final diagnosis:** CNS tuberculomas mimicking multiple brain metastases.

**Clinical pearl:** In TB-endemic regions, multifocal brain lesions in older patients may represent tuberculomas rather than metastases. Fundus examination can provide crucial diagnostic clues.

### Case 4 – Not all hemiparesis is vascular

A 73-year-old man, a chronic smoker and alcoholic, was referred from a private hospital after being thrombolysed for presumed acute ischemic stroke. His wife reported fever, headache, and vomiting for one week, followed by progressive left-sided weakness over three days, culminating in profound hemiparesis on the day of admission.

The private hospital CT reportedly showed a right parietal hypodensity interpreted as an evolving infarct, leading to thrombolysis. CT brain at our centre revealed a right parieto-occipital hypodense lesion, atypical for ischemic stroke. Given systemic symptoms, an infective cause was suspected. Mannitol and dexamethasone were initiated, and a lumbar puncture performed [13], [14].

CSF analysis on Day 3 showed lymphocytic pleocytosis, elevated ADA (22 U/L), and low glucose (42 mg/dL). CBNAAT was positive for *Mycobacterium tuberculosis*. MRI revealed a ring-enhancing lesion in the right paracentral lobule with surrounding edema, consistent with a tuberculoma. HIV testing was negative. Chest imaging showed no evidence of pulmonary TB.

ATT was started. Over the following week, he improved neurologically; on Day 30 he was discharged, ambulant with support. At 4-month follow-up, he continued to improve and was ambulant with minimal support.

**Final diagnosis:** CNS tuberculoma with tuberculous meningitis mimicking acute ischemic stroke.

**Clinical pearl:** Subacute hemiparesis with fever should not be labeled as stroke without CSF evaluation, especially in TB-endemic settings.

### Case 5 – Vanishing memory syndrome

A 29-year-old woman with no comorbidities presented with six months of progressive memory loss. Her family described increasing forgetfulness, difficulty recalling conversations, misplacing objects, and impaired daily functioning. There was no history of fever, headache, seizures, or neurological deficits. She also had no history of psychiatric illness, substance use, head trauma, metabolic disease, or family history of dementia. On admission, she was alert but disoriented to time, with impaired short-term memory and poor recall, though language and long-term memory were preserved [15], [16].

**Fig. 3:**
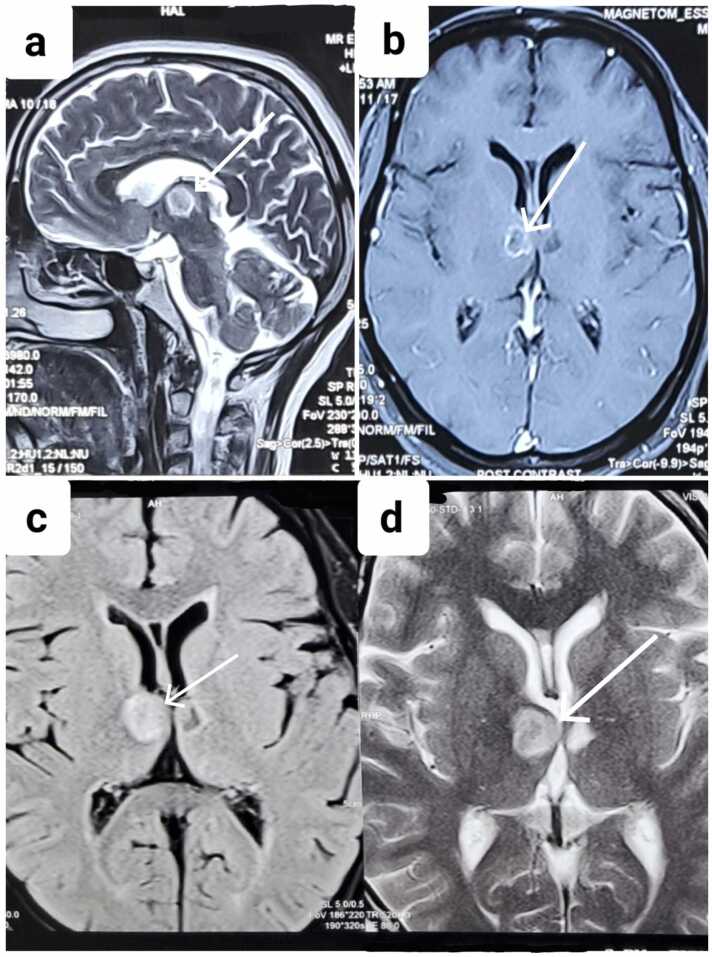
MRI features of thalamic tuberculoma of Case 5 (a) Sagittal T2-weighted image showing a hyperintense thalamic lesion (arrow). (b) Axial post-contrast T1-weighted image demonstrating ring enhancement of the thalamic lesion (arrow). (c) Axial FLAIR image showing hyperintense thalamic lesion with central hypointensity (arrow). (d) Axial T2-weighted image showing hyperintense thalamic lesion with perilesional edema (arrow).

CT scan done on Day 1 was non-contributory. Fundus examination was normal. Psychiatric evaluation was normal and did not account for her cognitive symptoms. CSF analysis on Day 2 revealed lymphocytic pleocytosis, elevated ADA (22 U/L), and low glucose (48 mg/dL); CBNAAT and culture were negative. MRI on Day 4 revealed a ring-enhancing lesion in the right thalamus with surrounding edema (Fig. 3a-d). HIV testing was negative. Chest imaging showed no evidence of pulmonary TB. Systemic evaluation showed no pulmonary or extrapulmonary TB.

She was started on mannitol and dexamethasone on Day 2, and ATT on Day 3. Over the next week, her memory and orientation improved, and by Day 10 her family noted clear progress. At three months, she was independent in daily activities and had no new neurological deficits.

## Differential diagnoses considered

1. Early-onset degenerative dementia
2. Autoimmune/paraneoplastic encephalitis
3. Thalamic stroke
4. Nutritional deficiency (B12, thiamine)
5. Metabolic/toxic causes
6. Primary psychiatric disorders (depression/pseudodementia)

**Final diagnosis:** Right thalamic tuberculoma presenting as isolated anterograde amnesia.

**Clinical pearl:** In young patients with isolated subacute memory loss, CNS TB should be considered, as thalamic tuberculomas can mimic degenerative or vascular disorders.

### Case 6 – Entered as epilepsy, exited as TB

A 42-year-old man, diabetic, chronic alcoholic and smoker, with longstanding epilepsy since age 22 without an identifiable cause and maintained on phenytoin 100 mg twice daily with recurrent breakthrough seizures, with no previous history of tuberculosis, presented with recurrent generalized tonic–clonic seizures over 24 h and five days of fever. At the referring hospital, his condition was attributed to breakthrough epilepsy [17], [18].

On admission, he was drowsy but arousable, with no focal deficits. CT brain showed multiple hypodense cortical and subcortical lesions, some with ill-defined margins and perilesional edema. Mannitol was started, and antiepileptics optimized.

**Fig. 4:**
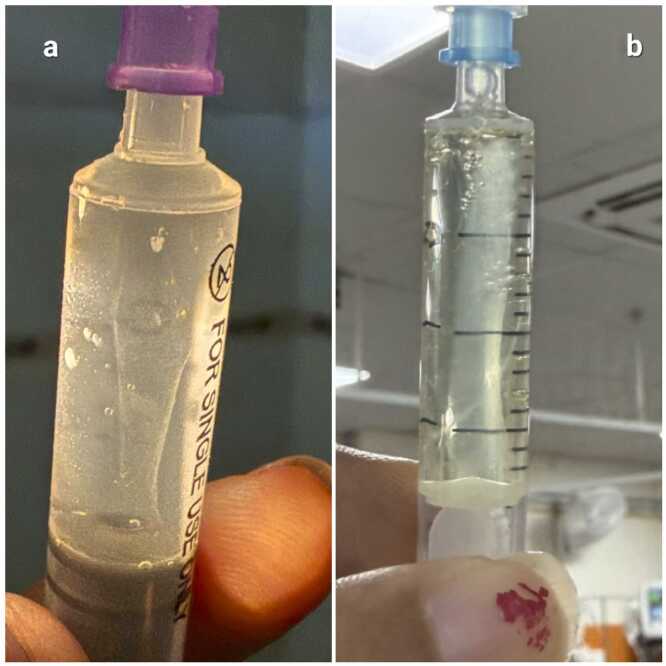
Photographs of cerebrospinal fluid (CSF) demonstrating the formation of a "cobweb coagulum." The presence of this delicate, web-like fibrinous clot is a classic sign suggestive of tuberculous meningitis. (a) cobweb coagulum from Case 1. (b) cobweb coagulum from Case 6.

CSF analysis on Day 2 revealed lymphocytic pleocytosis, low glucose (62 mg/dL), random blood sugar (RBS) at the time of lumbar puncture: 203 mg/dL, ADA 15 U/L, and a cobweb coagulum (Fig. 4b) suggestive of tuberculous meningitis. CBNAAT and culture were negative. Anti-tubercular therapy (ATT) and dexamethasone were started on Day 3. MRI on Day 5 demonstrated multiple ring-enhancing tuberculomas in bilateral frontal and parietal lobes. HIV testing was negative. Chest imaging showed no evidence of pulmonary TB.

He was treated with dexamethasone, ATT, antiepileptics and mannitol. Over the next week, his seizures were controlled, fever subsided, and sensorium improved. By Day 10, he was seizure-free and fully oriented. He was discharged on ATT, tapering steroids, and antiepileptics, with counselling on adherence and alcohol cessation. At one-month follow-up, he was seizure-free and cognitively stable.

**Final diagnosis:** CNS tuberculomas with tuberculous meningitis presenting as recurrent seizures in a patient with epilepsy.

**Clinical pearl:** Systemic features accompanying seizures in an epileptic patient should prompt evaluation for CNS tuberculosis rather than attributing all events to primary epilepsy.

**Table 1:** CNS tuberculosis case comparison.

Parameter	Case 1	Case 2	Case 3	Case 4	Case 5	Case 6
**Age (years) / Sex**	13 F	22 F	61 M	73 M	29 F	42 M
**Key Clinical Presentation**	Fever (4 months), headache, diplopia (left LR palsy)	New seizures during ATT for PTB; pre-existing epilepsy	Headache, vomiting, altered sensorium	Left hemiparesis after fever & headache; initially thrombolysed	Progressive memory loss (6 months)	Recurrent GTCS + fever; longstanding epilepsy
**Fundus Findings**	Bilateral choroidal tubercles	Normal	Bilateral choroidal tubercles	Normal	Normal	Normal
**CSF – Cells**	Lymphocytic pleocytosis	Lymphocytic pleocytosis	Lymphocytic pleocytosis	Lymphocytic pleocytosis	Lymphocytic pleocytosis	Lymphocytic pleocytosis
**CSF – Glucose (mg/dL)**	37	46	42	42	48	62
**CSF – ADA (U/L)**	19.2	16	18	22	22	15
**CSF – CBNAAT**	Negative	Negative	Negative	Positive	Negative	Negative
**CSF – Culture**	Negative	Negative	Negative	Negative	Negative	Negative
**HIV Status**	Negative	Negative	Negative	Negative	Negative	Negative
**Imaging Findings**	Multiple ring-enhancing lesions (brain & spine); MRS: ↑choline, ↑lipid	Multiple ring-enhancing tuberculomas (right parietal)	Multiple ring-enhancing lesions with edema	Ring-enhancing lesion (right paracentral lobule) with edema	Ring-enhancing lesion (right thalamus) with edema	Multiple bilateral frontal & parietal ring-enhancing tuberculomas with edema
**Treatment Given**	ATT + steroids + ICP management	ATT + steroids + optimized antiepileptics	ATT + steroids + ICP management	ATT + steroids + ICP management	ATT + steroids + ICP management	ATT + steroids + optimized antiepileptics
**Stage of Treatment Initiation**	Empirical after fundus/CSF (Day 3)	After CSF & MRI confirmation (Day 3)	After CSF results (Day 3)	After LP & CBNAAT positive (Day 3)	After CSF & MRI confirmation (Day 3)	After MRI & CSF confirmation (Day 3)
**Outcome**	Died (acute herniation, Day 23)	Improved; seizure-free at discharge	Improved; discharged stable	Significant recovery; ambulant with support	Marked memory improvement; independent	Seizure-free; improved sensorium

## Discussion

### Protean clinical mimicry

CNS tuberculosis often mimics diverse neurological diseases. In this series, patients were initially suspected to have neurocysticercosis, ischemic stroke, metastatic brain disease, epilepsy, or degenerative memory disorder. This clinical diversity complicates diagnosis in endemic regions.

## Diagnostic challenges in LMIC settings

MRI and MR spectroscopy are the gold standards for characterizing tuberculomas, but access is frequently delayed. A stepwise, resource-adapted approach proved effective:

• **CT brain** as the first-line screening tool.
• **Fundus examination**, which identified choroidal tubercles in two cases.
• **Lumbar puncture**, with classical CSF features (lymphocytic pleocytosis, low glucose, elevated ADA, cobweb coagulum) supporting TB even when CBNAAT was negative.
• **Exclusion of mimics** using targeted tests (e.g., *Taenia solium* IgG, systemic malignancy work-up).

Drug susceptibility testing could not be performed in most cases due to negative CSF cultures, which is common in CNS tuberculosis.

This structured algorithm (Fig. 5a) allowed timely initiation of therapy, even before advanced imaging or microbiological confirmation.

**Fig. 5a:**
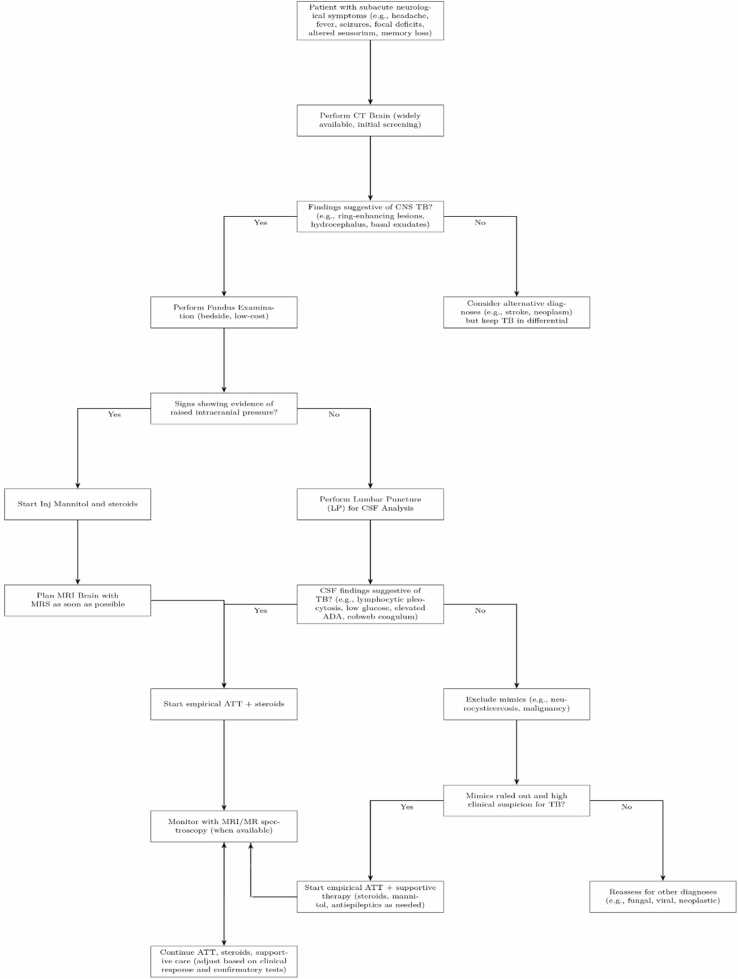
Proposed bedside diagnostic algorithm for central nervous system tuberculosis in low- and middle-income countries. The flowchart outlines a stepwise evaluation starting with CT brain (to exclude mass lesions), followed by fundus examination (to detect choroidal tubercles), lumbar puncture with CSF analysis, exclusion of mimics such as neurocysticercosis or malignancy, and early initiation of empirical anti-tubercular therapy where appropriate.

## Importance of early treatment

Outcomes correlated closely with timing of ATT initiation. Patients treated within 3–4 days of admission showed favourable recovery, whereas delays beyond Day 5, as in Case 1, were associated with death. This reflects the British Medical Research Council (MRC) staging system, in which advanced stage is associated with higher mortality (Table 2).

**Table 2:** Stages of Neurotuberculosis and Associated Mortality (Modified MRC Classification).

**Stage**	**Key Clinical Features**	**GCS**	**Mortality in HIV –ve if ATT started now**	**Mortality in HIV +ve if ATT started now**
**Prodrome (Preclinical)**	Low-grade fever, malaise, fatigue, weight loss, respiratory symptoms (children)	NA	–	–
**Stage 1 (Early TBM)**	Fever, headache, vomiting; normal consciousness; subtle/absent meningeal signs	15	∼20 %	∼40 %
**Stage 2 (Intermediate TBM)**	Confusion (GCS 10–14), focal neurological deficits, obvious meningeal signs	10–14	∼30 %	∼50 %
**Stage 3 (Advanced TBM)**	Coma (GCS <10), hemiparesis/quadriparesis, raised ICP, brainstem involvement, respiratory distress	< 10	> 60 %	∼75 %

This emphasizes a key principle: in LMICs, early empirical initiation of ATT may save lives, whereas waiting for definitive proof can be fatal.

## Treatment regimen

All patients received the WHO-recommended CNS tuberculosis regimen (2HRZE/10HR), with dosing as follows: isoniazid 10 mg/kg/day, rifampicin 10 mg/kg/day, pyrazinamide 30–35 mg/kg/day, and ethambutol 20 mg/kg/day.

All patients also received adjunctive dexamethasone, initiated at 0.4 mg/kg/day with a structured 6–8-week taper, as recommended in major TBM trials and international guidelines [19].

## Lessons for clinicians

1. **Think TB early** in any subacute neurological illness with systemic features.
2. **Utilize inexpensive tools** such as fundus examination and CSF analysis.
3. **Do not delay ATT** awaiting microbiological confirmation given its low sensitivity.
4. **Resource-adapted algorithms** can safely guide management where MRI or biopsy are inaccessible.

We propose a stepwise diagnostic and management algorithm for LMIC settings (Fig. 5a), complemented by a broader overview pathway (Fig. 5b).

**Fig. 5b:**
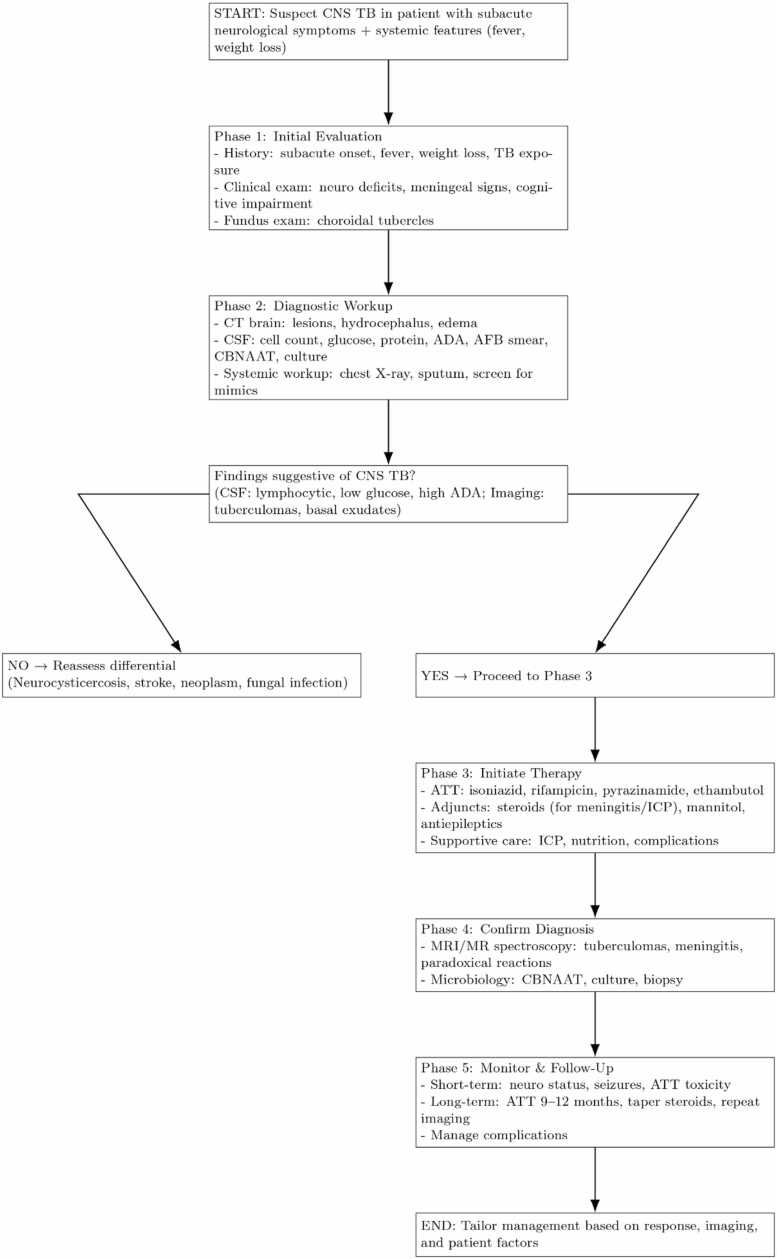
Clinical management pathway for CNS tuberculosis, integrating early clinical suspicion, supportive therapy (osmotic agents, corticosteroids, seizure control), and prompt anti-tubercular therapy initiation. The algorithm emphasizes pragmatic use of available diagnostics in resource-constrained settings.

## Global relevance

Although drawn from South India, these lessons extend to TB-endemic regions across Asia, Africa, and Latin America. In such settings, the cost of overtreatment is outweighed by the risk of fatal delay. Wider access to point-of-care CSF diagnostics and structured clinical pathways may help bridge the gap between suspicion and confirmation.

## Conclusion

This series highlights the deceptive nature of CNS TB, which may present as stroke, epilepsy, tumor, or cognitive decline. Early recognition demands high suspicion, bedside vigilance, and timely lumbar puncture, even when MRI or culture confirmation is delayed.

## CRediT authorship contribution statement

**K Sivakumar:** Writing – review & editing, Supervision, Project administration. **Parthiban Palaniappan:** Writing – original draft, Visualization, Investigation, Formal analysis, Data curation, Conceptualization.

## Consent for publication

Written informed consent for publication of clinical details and anonymised images was obtained from all patients (or their legal guardians in the case of minors). Copies of the consent forms are available to the Editor upon request.

## Ethics approval and consent to participate

Institutional ethics approval was not required for this case series as per local regulations, and a waiver was obtained.

## Ethics & Consent

Institutional ethics approval was not required for this case series as per local regulations, and a waiver was obtained.

Written informed consent for publication of clinical details and anonymised images was obtained from all patients (or their legal guardians for minors).

## Author agreement

None.

## Originality & Exclusivity

This manuscript is original, has not been published previously, and is not under consideration elsewhere.

## Authorship & Approval

• All authors listed have made substantial contributions to the work.
• All authors have read and approved the final version of the manuscript and agree to its submission.
• The order of authorship has been approved by all authors.

## Corresponding Author/ Guarantor

Dr. Parthiban Palaniappan is the corresponding author and guarantor, accepting full responsibility for the integrity of the work.

## Copyright/ License

Upon acceptance, the authors agree to assign copyright or grant Elsevier a publishing license as required.

## Guarantor

Dr. Parthiban Palaniappan is the guarantor of this article and accepts full responsibility for the integrity of the work.

## Reporting guideline compliance

This case series has been reported in accordance with the CARE guidelines.

## Funding

No external funding was received for this study.

## Declaration of Competing Interest

The authors declare that they have no known competing financial interests or personal relationships that could have appeared to influence the work reported in this paper.

## Data Availability

The datasets generated or analysed during this study are available from the corresponding author upon reasonable request.
